# Nucleosomes are enriched at the boundaries of hypomethylated regions (HMRs) in mouse dermal fibroblasts and keratinocytes

**DOI:** 10.1186/1756-8935-7-34

**Published:** 2014-12-02

**Authors:** Ximiao He, Raghunath Chatterjee, Desiree Tillo, Andrew Smith, Peter FitzGerald, Charles Vinson

**Affiliations:** Laboratory of Metabolism, National Cancer Institute, National Institutes of Health, 37 Convent Drive, Bethesda, MD 20892 USA; Human Genetics Unit, Indian Statistical Institute, 203 B. T. Road, Kolkata, 700108 India; Molecular and Computational Biology, University of Southern California 1050 Childs Way, Los Angeles, California 90089 USA; Genome Analysis Unit, Genetics Branch, National Cancer Institute, National Institutes of Health, 37 Convent Drive, Bethesda, MD 20892 USA

**Keywords:** CG methylation, Hypomethylated regions, HMR, Nucleosomes, Epigenomics, Keratinocytes, Fibroblasts

## Abstract

**Background:**

The interplay between epigenetic modifications and chromatin structure are integral to our understanding of genome function. Methylation of cytosine (5mC) at CG dinucleotides, traditionally associated with transcriptional repression, is the most highly studied chemical modification of DNA, occurring at over 70% of all CG dinucleotides in the genome. Hypomethylated regions (HMRs) often occur in CG islands (CGIs), however, they also occur outside of CGIs and function as cell-type specific enhancers. During the process of differentiation, reorganization of chromatin and nucleosome arrangement at regulatory regions is thought to occur in order for the establishment of cell-type specific transcriptional programs. However, the specifics regarding the organization of nucleosomes at HMRs and the potential mechanisms regulating nucleosome occupancy in these regions are unknown. Here, we have investigated nucleosome organization around hypomethylated regions (HMRs) identified in two mouse primary cells.

**Results:**

Microccocal nuclease (MNase) digested mononucleosomes from primary cultures of new-born female mouse dermal fibroblasts and keratinocytes were mapped and compared to the HMRs obtained from single base-pair resolution methylomes. In both cell types, we find that nucleosomes are enriched at HMR boundaries. In contrast to the nucleosomes found at boundaries of HMRs in CGIs, HMRs outside of CGIs are calculated to be preferentially bound by nucleosomes, with phased nucleosomes propagating into the methylated region. Nucleosomes are enriched at the tissue-specific HMRs (TS-HMR) boundaries in both cell types suggesting that nucleosome organization surrounding HMR boundaries is independent of methylation status. In addition, we find potential transcription factor (TF) binding sites (E-box motifs) enriched in non-CGI TS-HMR boundaries.

**Conclusions:**

Our results show that intrinsic nucleosome occupancy score (INOS) positively correlate with the nucleosome organization surrounding non-CGI TS-HMRs, suggesting that DNA sequence plays a role in the establishment of HMRs in the genome. Since nucleosomes impact all processes involving the genome, our results provide a link between epigenetic modifications, chromatin structure, and regulatory function.

**Electronic supplementary material:**

The online version of this article (doi:10.1186/1756-8935-7-34) contains supplementary material, which is available to authorized users.

## Background

Covalent modification of different bases of DNA occurs throughout genomes [1, 2]. In mammals, the typical DNA modification is methylation of cytosine in the CG dinucleotide with over 70% of CG dinucleotides being methylated [3–5]. Several single nucleotide resolution methylation maps show that unmethylated cytosines occur in clusters representing 2% to 3% of the genome, mainly in the CG rich regions termed CG islands (CGIs), which encompass 1% of the genome. Approximately 70% of promoters contain a CGI, and these promoters tend to regulate housekeeping genes [6], and the hypomethylation of these regions is critical for cellular function [4, 7, 8]. However, a number of hypomethylated regions (HMRs) exist outside of CGIs (non-CGI HMRs) and promoters, and these tend to vary across cell types and tissues [4, 7–15], suggestive of a possible regulatory role for non-CGIs as enhancer elements. Indeed, non-CGI HMRs have been shown to be enriched for transcription factor binding sites (TFBSs) [14], and are associated with expression of nearby tissue-specific genes [4, 8, 16, 17], indicating that hypomethylation of non-CGI regions is a hallmark of regulatory function.

In addition to the methylation status of regulatory elements, the primary organizational unit of chromatin, the nucleosome, is an important factor impacting the regulatory capacity of the genome. Nucleosomes restrict access to factors requiring the DNA template and nucleosome loss, depletion, or rearrangement is indicative of transcriptional activity in regulatory regions including promoters and enhancers across species [18, 19]. Moreover, the nucleosome itself is an important factor signaling regulatory activity. For example, nucleosomes harboring specific post-translational modifications have been shown to associate with active chromatin (for example, monomethylation of lysine 4 of histone H3 is enriched in nucleosomes flanking active or poised enhancers [20]). Nucleosomes have also been implicated in the targeting of DNA methylation, as they have also been shown to serve as tethering sites for DNA demethylases that, in turn, contribute to the formation of repressive chromatin states [21, 22].

Several studies have begun to investigate the relationship between DNA methylation and nucleosome organization. Many studies focus on the effects of DNA methylation on nucleosome stability. It has been demonstrated that CG methylation is reduced by the presence of nucleosomes [23–27], however, methylation is enriched within nucleosome core DNA *in vivo* [28]. Thus, the role of nucleosome positioning on DNA methylation as well as the effects of methylation on nucleosome positioning are not resolved, and it has been suggested that both can influence each other [29]. Recent studies focusing on specific regulatory regions (promoters and distal enhancers) indicate that nucleosome reorganization and depletion generally accompanies demethylation of regulatory elements leading to activation of these regions *in vivo* [29–31]. It has recently been shown that nucleosomes tend to be highly organized over regions that are differentially methylated between tissues even when these regions are not active [30].

Here, we have investigated nucleosome organization at the boundaries of HMRs where the DNA methylation transition occurs. To this end, we have generated genome-wide nucleosome maps produced by MNase digestion of nucleosomal DNA obtained from primary tissues from mouse fibroblasts and keratinocytes, followed by high-throughput sequencing. We next compared the nucleosome organization surrounding HMRs that we have recently identified in both cell types [5, 32] and found that nucleosomes are enriched at the HMR boundaries. We find that nucleosome organization at non-CGI HMR boundaries, which tend to be tissue-specific, is independent of methylation status. In addition, in contrast to HMRs in CGIs, nucleosomes at the boundaries of non-CGI HMRs are predicted by a model of intrinsic nucleosome occupancy [33], suggesting that nucleosomes are localized at their preferred sites, implicating a role for DNA sequence in demarcating these putative regulatory regions genome-wide.

## Results

### Genome-wide nucleosome maps of mouse dermal fibroblasts and keratinocytes

Micrococcal nuclease (MNase)-digested nucleosomal DNA from primary cultures of new-born female mouse fibroblasts and keratinocytes was sequenced using an Illumina HiSeq sequencer [34–38]. The resulting reads, mapped to the mouse genome, allowed the generation of map of nucleosome occupancy at high (27-35X) coverage for each cell type (Additional file 1: Table S1, Additional file 2: Figure S1).

As validation of the quality of these data, we assessed the nucleosome occupancy surrounding the transcription start site (TSS). We subdivided mouse promoters based on the presence of CGIs (+/-CGI) and their methylation status [5, 32]. We find that unmethylated promoters (+/-CGI) in both tissues are characterized by a nucleosome-depleted region upstream of the TSS, with phased nucleosomes (165 base pairs (bps) periodicity) occurring towards the body of the gene (Additional file 2: Figure S2). The degree of nucleosome depletion upstream of the TSS as well as the strength of the phasing is positively correlated with transcription, as previously observed in yeast [38, 39] and vertebrates [40–42]. The next largest group, are promoters that are methylated but do not contain a CGI (-HMR/-CGI: 8,803/28,283). In contrast to the previous groups, promoters of the most highly expressed genes in this class do not have a nucleosome depleted region or the phased nucleosome pattern similar to Saga-containing promoters in yeast [43] (Additional file 2: Figure S2).

### Nucleosomes localize at boundaries of hypomethylated regions of keratinocytes and fibroblasts

**Figure 1:**
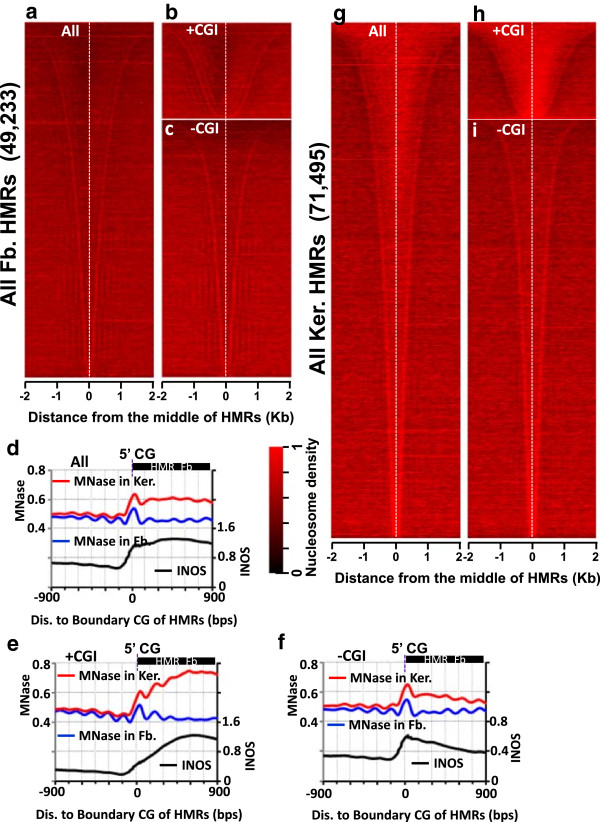
**Patterns of nucleosome occupancy at hypomethylated regions (HMRs) of mouse fibroblasts and keratinocytes. (a-c)** Heatmap of nucleosome densities at HMRs sorted by length in fibroblasts (Fb.) for **(a)** all HMRs, **(b)** HMRs overlapping with CGIs and **(c)** HMRs not overlapping with CGIs. **(d-f)** Average nucleosome occupancy measured in fibroblasts (blue) and keratinocytes (red) and intrinsic nucleosome occupancy scores (INOS, black) for **(d)** all fibroblast HMRs, **(e)** fibroblast HMRs overlapping CGIs (+CGIs), and **(f)** non-CGI containing fibroblast HMRs (-CGIs). HMRs are aligned by the first (5′) unmethylated CG dinucleotides. **(g-i)** same as in **(a-c)** but for mouse keratinocytes (Ker).

We next investigated the *in vivo* nucleosome organization surrounding 49,233 fibroblast hypomethylated regions (HMRs) [5] and 71,495 keratinocyte HMRs [32]. One difficulty in such an analysis is the assignment of the boundaries of HMRs. These boundaries could occur at the first unmethylated CG dinucleotide of the HMR, the methylated CG directly upstream and downstream of the HMR, or somewhere between the two. To gain insight into nucleosome distributions across HMRs, we aligned HMRs either by the first/second unmethylated CG or by the adjacent methylated CG (Additional file 2: Figure S3 and S4) located on average 150 bps from the first unmethylated CG (Additional file 2: Figure S5a and b). We observe a weak averaged nucleosome profile when HMR boundaries are assigned using the adjacent methylated CGs (Additional file 2: Figures S3a, S4a-d). However, when HMR boundaries are set using the first unmethylated CG, we find a striking pattern in both cell types with nucleosomes enriched at the boundaries with a periodic array of nucleosomes propagating into the methylated region (Figure 1a, d, g and Additional file 2: Figure S3b). Similar results were obtained when defining the HMR boundaries using the second unmethylated CG (Additional file 2: Figures S3c, S4e-h), although this approach yielded a less periodic nucleosome signal outside of the HMR boundaries (Additional file 2: Figure S5c).

Since a substantial fraction of HMR regions found in our methylome maps do not overlap a CGI (72.5% of all fibroblast HMRs and 81.2% of all identified keratinocyte HMRs), we next divided the HMRs into two groups: those that overlap with CGIs (Figure 1b, e, h) and those that do not (Figure 1c, f, i). For fibroblast HMRs overlapping CGIs, nucleosomes localize to the unmethylated CG boundary with phased nucleosomes extending into the methylated region, with a nucleosome depleted region in the middle of the HMR (Figure 1e). This nucleosome depleted region is at the TSS of expressed genes in CGIs (Additional file 2: Figure S2). Indeed, 78% (10,529/13,520) (Additional file 2: Figure S6a) of fibroblast HMRs within CGIs contain TSSs and the observed nucleosome depletion is presumably caused by RNA Pol II and associated factors that preferentially associate with CGI promoters (Additional file 2: Figure S2) [42, 44–46]. The 13,460 keratinocyte HMRs in CGIs are primarily promoters with nucleosomes at the boundary (Additional file 2: Figure S6c), however, the interior of these HMRs are more occupied by nucleosomes than the corresponding group in fibroblasts (Figure 1e, h), potentially reflecting the changes observed in the terminal stages of epidermal differentiation [47, 48].

Most HMRs are not in CGIs and they have a similar nucleosome pattern at the boundaries (Figure 1c, f, i). The 35,713 fibroblast and 58,035 keratinocyte HMRs without CGIs have nucleosomes at the boundary with phased nucleosomes spreading into the adjacent methylated region. However, in contrast to the HMRs within CGIs, the nucleosomes at the boundary of non-CGI HMRs are predictable by a model of intrinsic nucleosome sequence preference (intrinsic nucleosome occupancy scores (INOS)) [33] suggesting that the localization of nucleosomes at these boundaries might be driven by DNA sequence (Figure 1f).

### Nucleosome localization at non-CGI HMR boundaries does not depend on methylation status

**Figure 2:**
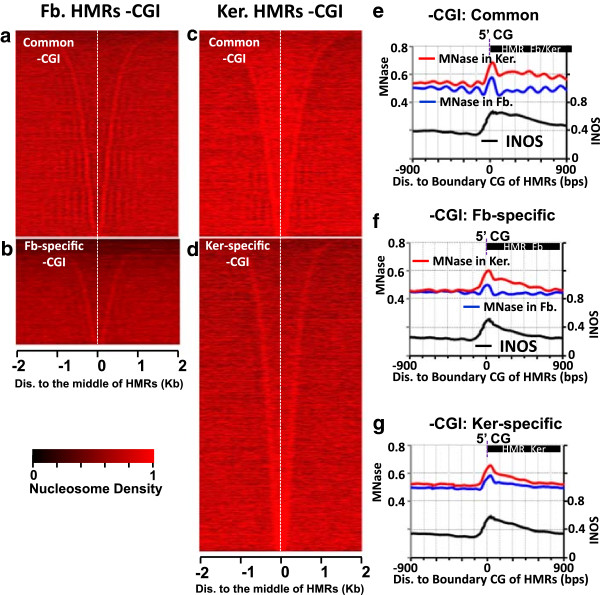
**Nucleosome organization in non-CGI HMRs is not dependent on methylation status. (a, b)** Heatmap of nucleosome density in fibroblasts (Fb.) surrounding non-CGI containing HMRs for **(a)** common (found at both keratinocytes and fibroblasts), and **(b)** fibroblast-specific HMRs. **(c, d)** Heatmap of nucleosome density surrounding non-CGI containing HMRs in keratinocyte (Ker.) for **(c)** common and **(d)** keratinocyte-specific HMRs. For all heatmaps, HMRs in each class are sorted by length. **(e-g)** Average nucleosome occupancy measured in fibroblasts (blue), and keratinocytes (red), and intrinsic nucleosome occupancy scores (INOS, black) surrounding the 5′ unmethylated CG of the HMR boundary for **(e)** common non-CGI HMRs, **(f)** fibroblast specific non-CGI HMRs, and **(g)** keratinocyte-specific non-CGI HMRs.

We next asked whether the observed nucleosome pattern in HMRs outside of CGIs is due to their methylation status. We subdivided HMRs into three groups: those that are common in both cell types and those that are differentially methylated between the two tissues. For the purposes of clarity, we will refer to these differentially methylated regions as either keratinocyte or fibroblast tissue-specific HMRs (TS-HMRs) to mean those HMRs that are found in only one of the two tissues compared in this study. In contrast to common HMRs, in which a substantial portion (36.3%) occurs within CGIs, TS-HMRs show only 0.4% overlap with CGIs. Common HMRs outside of CGIs have nucleosomes at their boundaries as predicted by INOS with one to three strongly phased nucleosomes extending into the methylated region (Figure 2a, c, e). A similar pattern is seen in the nucleosome occupancy profiles of TS-HMRs, albeit with weaker phasing surrounding the HMR (Figure 2b, d). The stronger phasing may be due to active transcription [49] as approximately 14% common non-CGI HMR contain a TSS (Additional file 2: Figure S6e). In contrast, 4% of TS-HMRs contain a TSS within 2 kbp of the HMR (Additional file 2: Figure S6b, d).

Cross-comparison of nucleosome occupancy profiles in differentially methylated regions between keratinocytes and fibroblasts shows a peak of nucleosome occupancy at the TS-HMR boundary in both cell types. This peak at the boundaries corresponds to regions of high INOS (Figure 2f, g), suggesting that DNA sequence encoded nucleosome occupancy and not methylation status is an important contributor to the observed nucleosome localization at non-CGI HMR boundaries.

### Nucleosome organization at extended HMRs

**Figure 3:**
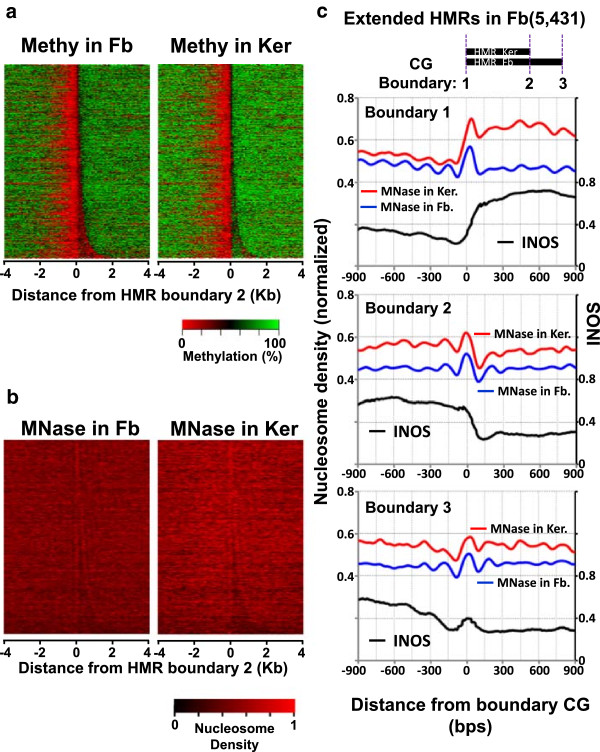
**Nucleosome organization at extended HMRs in fibroblasts. (a)** Heatmap of CG methylation in fibroblasts (Fb) and keratinocytes (Ker) for the common HMRs in which one boundary is identical, but the other boundary is extended in fibroblasts. The boundaries of these HMRs are grouped into three types: identical in two cells (boundary 1), not identical with short end (boundary 2), and with long end (boundary 3). These HMRs are aligned by boundary 2. **(b)** Heatmap of nucleosome positioning in fibroblasts and keratinocytes for the overlapping HMRs extended in fibroblasts aligned by boundary 2. **(c)** Average nucleosome density in fibroblasts (blue), keratinocytes (red), and intrinsic nucleosome occupancy scores (INOS, black) for each boundary type of the extended HMRs.

A common feature of HMRs shared between keratinocytes and fibroblasts is that the boundaries are not always identical. There exist 15,747 HMRs in which the one boundary is identical; however the other boundary is extended in one cell type [32]. The extended unmethylated region is on average 280 bps in length with a median of 170 bps (Figure 3a and b and Additional file 2: Figure S7a and b). The nucleosome localization at the common boundary of these extended HMRs is similar to the boundaries of those observed for HMRs with identical boundaries (Figure 3c and Additional file 2: Figures S7c, S8a and b). However for the extended HMRs, nucleosomes are displaced from their optimal positions at the identical boundary and phased nucleosomes extend into the methylated region, reflecting the influence of CGI-containing HMRs in the averaged profiles (2,453/7,497 or 32.7% of such HMRs overlap a CGI). The tissue-specific boundary of the shorter HMR (boundary 2) also has a positioned nucleosome and some modest phasing (Figure 3c and Additional file 2: Figure S7c). The extended boundary of the longer HMR (boundary 3) is reminiscent of boundaries in TS-HMRs with nucleosomes at the boundary, with little to no phasing of nucleosomes (Figure 3c and Additional file 2: Figure S7c). Nucleosomes centered on the boundary of both short and extended HMRs (boundaries 2 and 3) are in a region of high INOS, suggesting a prominent role for DNA sequence in specifying the boundaries of extended HMRs.

### Sequence features enriched at HMR boundaries

Our findings identify nucleosomes at boundaries of HMRs. However, the area under the ROC curve (AUC) score assessing the ability of a single CG dinucleotide to predict a nucleosome at the boundary of a HMR is modest (AUC =0.52, Additional file 2: Figure S9a). In contrast, the INOS calculation is more robust at predicting if a nucleosome will localize to these regions (AUC =0.79, Additional file 2: Figure S9a). Moreover, the predictive power of INOS is higher for the nucleosomes that are most highly enriched at HMR boundaries (that is, the top 20% of nucleosome peaks at boundaries, with an AUC =0.83 for INOS (AUC =0.81 for all the fibroblast-specific HMR boundaries), Additional file 2: Figure S9b), providing evidence that the broader sequence contexts in which these unmethylated CGs occur is important for the enrichment of nucleosomes at HMR boundaries.

**Figure 4:**
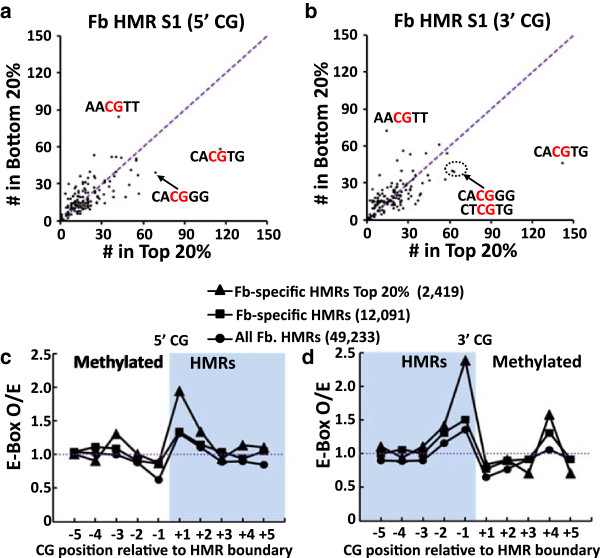
**E-box motifs are enriched at the boundaries of non-CGI HMRs. (a, b)** Comparison of 6-mer occurrences surrounding the **(a)** 5′ unmethylated CG and **(b)** 3′ unmethylated CG at the boundary of fibroblast-specific HMRs in the top 20% and bottom 20% of *in vivo* nucleosome peaks. (**c)** Ratio of observed/expected frequencies of the CACGTG pattern at -5 to +5 CGs for fibroblast HMRs (5′ CG): all HMRs (All, black circle), fibroblast-specific HMRs (black square) and fibroblast-specific HMRs with the top 20% *in vivo* nucleosome density at the boundary CG (Top 20%, black triangle). **(d)** Same as in **(c)** but at the 3′ boundary of fibroblast HMRs (3′ CG).

While INOS shows a general positive correspondence to the observed nucleosome occupancy at TS-HMR boundaries, this correlation is not perfect (Additional file 2: Figure S9). We therefore sought to identify additional DNA sequence features besides INOS that contribute to the observed nucleosome arrangements at HMR boundaries *in vivo*. We computed the enrichment of all 6-mers centered on the unmethylated CG at both HMR boundaries. Motifs enriched in the top 20% of all *in vivo* nucleosome peaks include GC rich 6-mers (CACGTG, CACGGG, CTCGTG) and motifs enriched at the HMR boundaries not bound by nucleosomes (bottom 20%) tend to be AT-rich (AACGTT, AACGTG) (Figures 4a and b, and Additional file 2: Figure S10a and b), consistent with base composition being a dominant contributor to INOS [33]. Interestingly, the E-box motif (CACGTG) is the most frequently occurring 6-mer at the unmethylated CG at the boundary at TS-HMRs that have high observed nucleosome occupancy in both cell types (Figure 4a and b and Additional file 2: Figure S10a and b) [7], being enriched approximately 2.0-fold at boundaries (Figure 4c and d and Additional file 2: Figure S10c and d). We also find that the E-Box motif is predicted to be well bound by nucleosomes and potentially its function is not as a TFBS but as a nucleosome binding site [50], as 150-bps sequences containing this motif in the mouse genome tend to be in sequences with higher than average INOS (INOS with E-box motif =0.59 vs. genome average =0.23). Taken together, this suggests that overlapping genomic signals, TFBS, and intrinsic nucleosome sequence preferences, contribute to the potential regulatory functions of TS-HMRs.

## Discussion

We have compared genome-wide nucleosome maps in mouse fibroblasts and keratinocytes with methylome data and have found that nucleosomes are enriched at the boundaries of HMRs. Furthermore, the methylation status of the HMR does not affect nucleosome localization at the boundary. The localization of nucleosomes at the boundaries of HMRs in CGIs is not predicted by INOS, indicating that additional biochemical forces are organizing HMR boundaries. Promoters in CGIs have a similar nucleosome organization to yeast, with a nucleosome-depleted region at the TSS and phased nucleosomes extending into the gene body [42]. However, the biochemical mechanisms that produce these positioned nucleosomes are different. In yeast, the nucleosome depleted promoter is AT rich and nucleosomes do not bind these sequences well. In contrast, mammalian promoters tend to be GC-rich and nucleosome depleted even though nucleosomes preferentially bind these sequences [45, 46]. Potentially, the DNA sequences that occur in CGIs [51] recruit remodelers [52] that are critical for the displacement of nucleosomes from favored binding positions. More impressively, in the case of HMRs outside of CGIs, which tend to be tissue-specific, we show that INOS explains a substantial fraction (AUC =0.81) of the nucleosome localization at boundaries, providing evidence that DNA sequence demarcates the boundaries of TS-HMRs in the genome. Sequence preferences of nucleosomes also play a role in the definition of the boundaries of extended HMRs, however, the exact mechanisms that regulate HMR length between tissue types warrant further investigation.

E-box motifs are enriched at boundaries of TS-HMRs in both cell types, particularly for HMRs with a nucleosome at the boundary. While some transcription factors, such as nuclear receptor proteins can bind nucleosomal DNA, B-HLH proteins cannot [50]. Nucleosome enrichment within HMR boundaries confirms and extends previous observations regarding the encoding of nucleosome occupancy over regulatory regions [45, 46]. Since many enhancers tend to be cell-type specific, high nucleosome occupancy of TFBS can restrict their utilization to a certain cell type. In addition, nucleosomes can reinforce and promote cooperative interactions between TFs in displacing nucleosomes from their preferred sites, providing higher specificity in gene regulation [53, 54]. The E-box motif is a sequence that nucleosome preferentially binds suggesting its presence at the HMR boundary is not to be a TFBS but a nucleosome localization site. Alternatively, the E-Box may function as both, sometimes being bound by a nucleosome and other times being bound by a B-HLH protein. Whether or not HLH proteins do in fact bind these E-Box sequences, suggesting equilibrium between a nucleosome and HLH protein binding and potential enhancer function of TS-HMRs, however, remains to be determined.

## Conclusions

We have mapped genome-wide nucleosome organization in two mouse primary tissue types, providing an important resource for the understanding of chromatin structure and gene regulation. Comparing these maps with HMRs obtained from single-base pair resolution methylomes has allowed the identification of the nucleosome arrangements in these regions. In particular, we find that nucleosomes are enriched at the boundaries of HMRs. Nucleosome organization at HMR boundaries is independent of methylation status. For HMRs not in CGI, boundaries are calculated to be well bound by nucleosome as occurs *in vivo*. Since hypomethylation of non-CGI regions is a hallmark of regulatory activity, our findings have important implications for the specification of chromatin architecture at regulatory regions in the genome.

## Methods

### Mouse primary keratinocytes and dermal fibroblasts

NIH research guidelines and IACUC approved animal study protocols were followed in this study. Keratinocytes and dermal fibroblasts were cultured from newborn wild type according to the protocol described previously [55]. Primary keratinocytes were seeded at a density of one mouse epidermis per 10 cm dish or equivalent in calcium and magnesium free SMEM (GIBCO Laboratories, Grand Island, NY, USA), supplemented with 8% Chelex (Bio-Rad, Richmond, CA, USA) treated FBS (Atlanta Biologicals, Inc.) and 0.2 mM calcium (CaCl2). Dermal fibroblasts were also seeded at a density of one mouse dermis per 10-cm-dish or equivalent in DMEM/F12: GlutaMAX medium (Invitrogen) with 10% FBS.

### Micrococcal nuclease (MNase) digestion and mapping of MNase-seq data

Primary cultures of female primary mouse dermal fibroblasts or keratinocytes were harvested and washed once with ice cold PBS. Resuspended cell pellet (approximately 10^8^ cells) were resuspended in 5 mL of ice cold NP-40 lysis buffer (10 mM Tris–HCl (pH 7.4), 10 mM NaCl, 3 mM MgCl_2_, 0.5% NP-40) and incubated for 5 min in ice. Nuclei were washed with MNase digestion buffer (10 mM Tris–HCl (pH 7.4), 15 mM NaCl, 60 mM KCl) and resuspended in MNase digestion buffer containing 1 mM CaCl2. Nuclei were digested with MNase for 10 and 15 min at 37 C. The MNase digestion was stopped by putting the samples on ice and adding 100 mM EDTA and 10 mM EGTA (pH 7.5). MNase digested DNA were purified with the Qiagen PCR purification kit after digestion with protease K (Qiagen). Isolated DNA was eluted in elution buffer (Qiagen). Mnase digested DNA fragments were separated on a 2% agarose gel. DNA corresponding to the mononucleosomal bands was gel extracted and pulled from all digestion. The libraries for sequencing were prepared according to the standard protocol for the Illumina HiSeq2000 sequencing platform with 102 bp paired end reads. Paired end reads of MNase-seq data were aligned using Novoalign software (http://www.novocraft.com/) with default parameters for both primary dermal fibroblasts and keratinocytes to the mouse genome (UCSC build mm9). Reads mapping to more than one location were discarded, and data were filtered to include only those sequences that had a mate pair match within 100 to 160 bp. Counts were recorded at the midpoint of the mate pair alignment, and Gaussian smoothing was applied to yield a continuous measure of nucleosome signal across the entire genome [56].

### Annotations

TSS information was extracted from RefGene annotations downloaded from the UCSC genome browser (https://genome.ucsc.edu/) for version of mm9. For genes with identical transcript start and stop sites, only one was retained. We downloaded the CG island annotations from the UCSC genome browser for version mm9 of the mouse genome.

### Female mouse primary dermal fibroblast and keratinocyte methylomes

The fibroblast methylome was produced and released by our group [5]. Generation of the genome-wide primary female mouse keratinocyte methylation data is described in the accompanied paper [32]. Regions of hypomethylation (hypomethylated regions (HMRs)) were defined using a two-state Hidden Markov Model (HMM) described in [7].

### Intrinsic nucleosome occupancy scores

We predicted intrinsic nucleosome occupancy scores (INOSs) across HMRs using the Lasso linear model described in [33]. For each HMR, we calculated the INOSs at every base pair using a sliding window of 147 bp.

### Data submission

Two biological replicates of MNase-seq for each primary dermal fibroblasts and keratinocytes are in the process of GEO submission. Keratinocyte methylome data have been submitted to the GEO database with accession number (GSE44918) [32]. Two biological replicates of keratinocyte mRNA-seq data are in the process of submission to the GEO database [32]. Fibroblast methylome and mRNA-seq data have been obtained from the GEO accession number (GSE44942) [5]. The second biological replicate RNA-seq data of dermal fibroblasts are in the process of submission to the GEO database [32]. All the sequencing data for fibroblasts and keratinocytes will be submitted to the GEO database with accession numbers (GSE44942 and GSE44918 respectively). The data can also be obtained from the authors upon request.

## Electronic supplementary material

Additional file 1: Table S1: Statistics on MNase-seq (102 bp paired end reads). (PDF 33 KB)

Additional file 2: Figure S1: Distribution of insert length of library of MNase-seq in fibroblasts and keratinocytes. **Figure S2.** Nucleosome occupancy at fibroblast and keratinocyte promoters. **Figure S3.** Nucleosome occupancy at fibroblast HMRs aligned using different CG dinucleotides to define HMR boundaries. **Figure S4.** Average *in vivo* and predicted nucleosome occupancy at the 5′ boundaries of fibroblast HMRs using different boundary CGs. **Figure S5.** Location and nucleosome periodicity of potential CG boundaries used to define HMRs. **Figure S6.** Locations of transcription start sites (TSS) relative to HMRs in keratinocytes and fibroblasts. **Figure S7.** Nucleosome organization at extended HMRs in keratinocytes. **Figure S8.** Average nucleosome density and INOS at common HMRs with identical boundaries (C1). **Figure S9.** ROC curve of two predictors of nucleosome occupancy in fibroblast. **Figure S10.** E-box motif enrichment at the boundaries of non-CGI HMRs in keratinocytes. (PDF 1 MB)

## References

[1] Bird AP, Taggart MH, Smith BA (1979). Methylated and unmethylated DNA compartments in the sea urchin genome. Cell.

[2] Jeltsch A (2010). Molecular biology. Phylogeny of methylomes. Science.

[3] Lister R, O’Malley RC, Tonti-Filippini J, Gregory BD, Berry CC, Millar AH, Ecker JR (2008). Highly integrated single-base resolution maps of the epigenome in Arabidopsis. Cell.

[4] Lister R, Pelizzola M, Dowen RH, Hawkins RD, Hon G, Tonti-Filippini J, Nery JR, Lee L, Ye Z, Ngo QM, Edsall L, Antosiewicz-Bourget J, Stewart R, Ruotti V, Millar AH, Thomson JA, Ren B, Ecker JR (2009). Human DNA methylomes at base resolution show widespread epigenomic differences. Nature.

[5] Mann IK, Chatterjee R, Zhao J, He X, Weirauch MT, Hughes TR, Vinson C (2013). CG methylated microarrays identify a novel methylated sequence bound by the CEBPB|ATF4 heterodimer that are active in vivo. Genome Res.

[6] Saxonov S, Berg P, Brutlag DL (2006). A genome-wide analysis of CpG dinucleotides in the human genome distinguishes two distinct classes of promoters. Proc Natl Acad Sci U S A.

[7] Molaro A, Hodges E, Fang F, Song Q, McCombie WR, Hannon GJ, Smith AD (2011). Sperm methylation profiles reveal features of epigenetic inheritance and evolution in primates. Cell.

[8] Stadler MB, Murr R, Burger L, Ivanek R, Lienert F, Scholer A, van Nimwegen E, Wirbelauer C, Oakeley EJ, Gaidatzis D, Tiwari VK, Schubeler D (2011). DNA-binding factors shape the mouse methylome at distal regulatory regions. Nature.

[9] Rakyan VK, Down TA, Thorne NP, Flicek P, Kulesha E, Graf S, Tomazou EM, Backdahl L, Johnson N, Herberth M, Howe KL, Jackson DK, Miretti MM, Fiegler H, Marioni JC, Birney E, Hubbard TJ, Carter NP, Tavare S, Beck S (2008). An integrated resource for genome-wide identification and analysis of human tissue-specific differentially methylated regions (tDMRs). Genome Res.

[10] Yagi S, Hirabayashi K, Sato S, Li W, Takahashi Y, Hirakawa T, Wu G, Hattori N, Ohgane J, Tanaka S, Liu XS, Shiota K (2008). DNA methylation profile of tissue-dependent and differentially methylated regions (T-DMRs) in mouse promoter regions demonstrating tissue-specific gene expression. Genome Res.

[11] Laurent L, Wong E, Li G, Huynh T, Tsirigos A, Ong CT, Low HM, Kin Sung KW, Rigoutsos I, Loring J, Wei CL (2010). Dynamic changes in the human methylome during differentiation. Genome Res.

[12] Krivega I, Dean A (2011). Enhancer and promoter interactions-long distance calls. Curr Opin Genet Dev.

[13] Meissner A, Mikkelsen TS, Gu H, Wernig M, Hanna J, Sivachenko A, Zhang X, Bernstein BE, Nusbaum C, Jaffe DB, Gnirke A, Jaenisch R, Lander ES (2008). Genome-scale DNA methylation maps of pluripotent and differentiated cells. Nature.

[14] Ziller MJ, Gu H, Muller F, Donaghey J, Tsai LT, Kohlbacher O, De Jager PL, Rosen ED, Bennett DA, Bernstein BE, Gnirke A, Meissner A (2013). Charting a dynamic DNA methylation landscape of the human genome. Nature.

[15] Schmidl C, Klug M, Boeld TJ, Andreesen R, Hoffmann P, Edinger M, Rehli M (2009). Lineage-specific DNA methylation in T cells correlates with histone methylation and enhancer activity. Genome Res.

[16] Aran D, Sabato S, Hellman A (2013). DNA methylation of distal regulatory sites characterizes dysregulation of cancer genes. Genome Biol.

[17] Taberlay PC, Kelly TK, Liu CC, You JS, De Carvalho DD, Miranda TB, Zhou XJ, Liang G, Jones PA (2011). Polycomb-repressed genes have permissive enhancers that initiate reprogramming. Cell.

[18] Han M, Grunstein M (1988). Nucleosome loss activates yeast downstream promoters in vivo. Cell.

[19] Wu C, Wong YC, Elgin SC (1979). The chromatin structure of specific genes: II. Disruption of chromatin structure during gene activity. Cell.

[20] Heintzman ND, Stuart RK, Hon G, Fu Y, Ching CW, Hawkins RD, Barrera LO, Van Calcar S, Qu C, Ching KA, Wang W, Weng Z, Green RD, Crawford GE, Ren B (2007). Distinct and predictive chromatin signatures of transcriptional promoters and enhancers in the human genome. Nat Genet.

[21] Jeong S, Liang G, Sharma S, Lin JC, Choi SH, Han H, Yoo CB, Egger G, Yang AS, Jones PA (2009). Selective anchoring of DNA methyltransferases 3A and 3B to nucleosomes containing methylated DNA. Mol Cell Biol.

[22] You JS, Kelly TK, De Carvalho DD, Taberlay PC, Liang G, Jones PA (2011). OCT4 establishes and maintains nucleosome-depleted regions that provide additional layers of epigenetic regulation of its target genes. Proc Natl Acad Sci U S A.

[23] Felle M, Hoffmeister H, Rothammer J, Fuchs A, Exler JH, Langst G (2011). Nucleosomes protect DNA from DNA methylation in vivo and in vitro. Nucleic Acids Res.

[24] Gowher H, Stockdale CJ, Goyal R, Ferreira H, Owen-Hughes T, Jeltsch A (2005). De novo methylation of nucleosomal DNA by the mammalian Dnmt1 and Dnmt3A DNA methyltransferases. Biochemistry.

[25] Jiang Y, Schneck JL, Grimes M, Taylor AN, Hou W, Thrall SH, Sweitzer SM (2011). Methyltransferases prefer monomer over core-trimmed nucleosomes as in vitro substrates. Anal Biochem.

[26] Kelly TK, Liu Y, Lay FD, Liang G, Berman BP, Jones PA (2012). Genome-wide mapping of nucleosome positioning and DNA methylation within individual DNA molecules. Genome Res.

[27] Takeshima H, Suetake I, Tajima S (2008). Mouse Dnmt3a preferentially methylates linker DNA and is inhibited by histone H1. J Mol Biol.

[28] Chodavarapu RK, Feng S, Bernatavichute YV, Chen PY, Stroud H, Yu Y, Hetzel JA, Kuo F, Kim J, Cokus SJ, Casero D, Bernal M, Huijser P, Clark AT, Kramer U, Merchant SS, Zhang X, Jacobsen SE, Pellegrini M (2010). Relationship between nucleosome positioning and DNA methylation. Nature.

[29] Portela A, Liz J, Nogales V, Setien F, Villanueva A, Esteller M (2013). DNA methylation determines nucleosome occupancy in the 5′-CpG islands of tumor suppressor genes. Oncogene.

[30] Taberlay PC, Statham AL, Kelly TK, Clark SJ, Jones PA (2014). Reconfiguration of nucleosome-depleted regions at distal regulatory elements accompanies DNA methylation of enhancers and insulators in cancer. Genome Res.

[31] Teif VB, Vainshtein Y, Caudron-Herger M, Mallm JP, Marth C, Hofer T, Rippe K (2012). Genome-wide nucleosome positioning during embryonic stem cell development. Nat Struct Mol Biol.

[32] Chatterjee R, He X, Huang D, FitzGerald P, Smith A, Vinson C (2014). High-resolution genome-wide DNA methylation maps of mouse primary female dermal fibroblasts and keratinocytes. Epigenetics Chromatin.

[33] Tillo D, Hughes TR (2009). G + C content dominates intrinsic nucleosome occupancy. BMC Bioinformatics.

[34] Axel R (1975). Cleavage of DNA in nuclei and chromatin with staphylococcal nuclease. Biochemistry.

[35] Jiang C, Pugh BF (2009). Nucleosome positioning and gene regulation: advances through genomics. Nat Rev Genet.

[36] Mavrich TN, Jiang C, Ioshikhes IP, Li X, Venters BJ, Zanton SJ, Tomsho LP, Qi J, Glaser RL, Schuster SC, Gilmour DS, Albert I, Pugh BF (2008). Nucleosome organization in the Drosophila genome. Nature.

[37] Shivaswamy S, Bhinge A, Zhao Y, Jones S, Hirst M, Iyer VR (2008). Dynamic remodeling of individual nucleosomes across a eukaryotic genome in response to transcriptional perturbation. PLoS Biol.

[38] Zhang Z, Wippo CJ, Wal M, Ward E, Korber P, Pugh BF (2011). A packing mechanism for nucleosome organization reconstituted across a eukaryotic genome. Science.

[39] Lee W, Tillo D, Bray N, Morse RH, Davis RW, Hughes TR, Nislow C (2007). A high-resolution atlas of nucleosome occupancy in yeast. Nat Genet.

[40] Deng T, Zhu ZI, Zhang S, Leng F, Cherukuri S, Hansen L, Marino-Ramirez L, Meshorer E, Landsman D, Bustin M (2013). HMGN1 modulates nucleosome occupancy and DNase I hypersensitivity at the CpG island promoters of embryonic stem cells. Mol Cell Biol.

[41] Sadeh R, Allis CD (2011). Genome-wide “re”-modeling of nucleosome positions. Cell.

[42] Schones DE, Cui K, Cuddapah S, Roh TY, Barski A, Wang Z, Wei G, Zhao K (2008). Dynamic regulation of nucleosome positioning in the human genome. Cell.

[43] Huisinga KL, Pugh BF (2004). A genome-wide housekeeping role for TFIID and a highly regulated stress-related role for SAGA in Saccharomyces cerevisiae. Mol Cell.

[44] Rozenberg JM, Shlyakhtenko A, Glass K, Rishi V, Myakishev MV, FitzGerald PC, Vinson C (2008). All and only CpG containing sequences are enriched in promoters abundantly bound by RNA polymerase II in multiple tissues. BMC Genomics.

[45] Tillo D, Kaplan N, Moore IK, Fondufe-Mittendorf Y, Gossett AJ, Field Y, Lieb JD, Widom J, Segal E, Hughes TR (2010). High nucleosome occupancy is encoded at human regulatory sequences. PLoS One.

[46] Valouev A, Johnson SM, Boyd SD, Smith CL, Fire AZ, Sidow A (2011). Determinants of nucleosome organization in primary human cells. Nature.

[47] Botchkarev VA, Gdula MR, Mardaryev AN, Sharov AA, Fessing MY (2012). Epigenetic regulation of gene expression in keratinocytes. J Invest Dermatol.

[48] Gdula MR, Poterlowicz K, Mardaryev AN, Sharov AA, Peng Y, Fessing MY, Botchkarev VA (2013). Remodeling of three-dimensional organization of the nucleus during terminal keratinocyte differentiation in the epidermis. J Invest Dermatol.

[49] Schlesinger F, Smith AD, Gingeras TR, Hannon GJ, Hodges E (2013). De novo DNA demethylation and non-coding transcription define active intergenic regulatory elements. Genome Res.

[50] He X, Chatterjee R, John S, Bravo H, Sathyanarayana BK, Biddie SC, FitzGerald PC, Stamatoyannopoulos JA, Hager GL, Vinson C (2013). Contribution of nucleosome binding preferences and co-occurring DNA sequences to transcription factor binding. BMC Genomics.

[51] FitzGerald PC, Shlyakhtenko A, Mir AA, Vinson C (2004). Clustering of DNA sequences in human promoters. Genome Res.

[52] Yen K, Vinayachandran V, Batta K, Koerber RT, Pugh BF (2012). Genome-wide nucleosome specificity and directionality of chromatin remodelers. Cell.

[53] Mirny LA (2010). Nucleosome-mediated cooperativity between transcription factors. Proc Natl Acad Sci U S A.

[54] Polach KJ, Widom J (1996). A model for the cooperative binding of eukaryotic regulatory proteins to nucleosomal target sites. J Mol Biol.

[55] Rishi V, Bhattacharya P, Chatterjee R, Rozenberg J, Zhao J, Glass K, Fitzgerald P, Vinson C (2010). CpG methylation of half-CRE sequences creates C/EBPalpha binding sites that activate some tissue-specific genes. Proc Natl Acad Sci U S A.

[56] Ranjan A, Mizuguchi G, FitzGerald PC, Wei D, Wang F, Huang Y, Luk E, Woodcock CL, Wu C (2013). Nucleosome-free region dominates histone acetylation in targeting SWR1 to promoters for H2A.Z replacement. Cell.

