# Supplementary material for: Nucleosomes are enriched at the boundaries of hypomethylated regions (HMRs) in mouse dermal fibroblasts and keratinocytes
Source: Epigenetics Chromatin. 2014 Dec 2;7:34. doi: 10.1186/1756-8935-7-34 (PMC4265496; doi:10.1186/1756-8935-7-34)
Supplement: Supplementary file 1 — Additional file 1: Table S1: Statistics on MNase-seq (102 bp paired end reads). (PDF 33 KB) [file 13072_2014_341_MOESM1_ESM.pdf]

Table S1. MNase-seq (102-bp paired end reads)

	#Total reads	#Aligned reads	Fold coverage
**Fibroblasts**	1,275,365,148	922,675,745	35.4
**Keratinocytes**	1,225,224,534	703,528,851	27.0
