# Supplementary material for: Nucleosomes are enriched at the boundaries of hypomethylated regions (HMRs) in mouse dermal fibroblasts and keratinocytes
Source: Epigenetics Chromatin. 2014 Dec 2;7:34. doi: 10.1186/1756-8935-7-34 (PMC4265496; doi:10.1186/1756-8935-7-34)
Supplement: Supplementary file 2 — Additional file 2: Figure S1: Distribution of insert length of library of MNase-seq in fibroblasts and keratinocytes. Figure S2. Nucleosome occupancy at fibroblast and keratinocyte promoters. Figure S3. Nucleosome occupancy at fibroblast HMRs aligned using different CG dinucleotides to define HMR boundaries. Figure S4. Average in vivo and predicted nucleosome occupancy at the 5′ boundaries of fibroblast HMRs using different boundary CGs. Figure S5. Location and nucleosome periodicity of potential CG boundaries used to define HMRs. Figure S6. Locations of transcription start sites (TSS) relative to HMRs in keratinocytes and fibroblasts. Figure S7. Nucleosome organization at extended HMRs in keratinocytes. Figure S8. Average nucleosome density and INOS at common HMRs with identical boundaries (C1). Figure S9. ROC curve of two predictors of nucleosome occupancy in fibroblast. Figure S10. E-box motif enrichment at the boundaries of non-CGI HMRs in keratinocytes. (PDF 1 MB) [file 13072_2014_341_MOESM2_ESM.pdf]

Additional file 2:

Figure legends:

Figure S1. Distribution of insert length of library of MNase-seq in fibroblasts and keratinocytes. The distribution of the insert length of the paired-reads in MNase-seq library in fibroblasts and keratinocytes. MNase-seq data for fibroblast (Fb) in blue and keratinocytes (Ker) in red.

Figure S2. Nucleosome occupancy at fibroblast and keratinocyte promoters. **(a-d)** Heatmap of nucleosome density for +/-2 kb surrounding the transcription start site in fibroblasts. Promoters were divided into four groups: +/- HMR, +/- CGI and are grouped by mRNA expression levels using RPKM (Reads Per Kilobase per Million mapped reads). **(e-h)** Same as in **(a-d)**, but keratinocyte nucleosome density is shown.

Figure S3. Nucleosome occupancy at fibroblast HMRs aligned using different CG dinucleotides to define HMR boundaries. Heatmap of nucleosome densities at all HMRs sorted by length in fibroblasts (Fb), in which HMR boundaries are defined using the **(a)** first methylated CpG dinucleotide (mCG), **(b)** first unmethylated CpG (uCG), and **(c)** second unmethylated CG (uCG).

Figure S4. Average *in vivo* and predicted nucleosome occupancy at the 5′ boundaries of fibroblast HMRs using different boundary CGs. **(a-d)** Average nucleosome density and intrinsic nucleosome occupancy scores (INOS) surrounding the first methylated CpG (mCG) for **(a)** fibroblast (Fb.) HMRs overlapping with CGIs, **(b)** non-CGI Fb. HMRs (**c**) Fb-specific HMRs outside of CGIs, and **(d)** common non-CGI HMRs. **(e-h)** Same as in **(a-d)**, but using the second unmethylated CG (second uCG).

Figure S5. Location and nucleosome periodicity of potential CG boundaries used to define HMRs. **(a)** Location of CG dinucleotides used to define the boundaries of fibroblast HMRs for the heatmaps in **Figure S3.** First methylated CpG (mCG) in black, first unmethylated CpG (uCG) in red, and second uCG in blue. **(b)** Average distances between mCGs, first uCG and second uCG. **(c)** Autocorrelation plots of fibroblast nucleosome occupancy data extending away from the 5′ HMR boundaries in fibroblasts. The y-axis on each plot shows the Pearson correlation values obtained from correlating the nucleosome occupancy data from the 5′ boundary of the

HMR with shifted versions of itself from 0 to 900 bp upstream of this boundary defined by either the first uCG (red) or the second uCG (blue).

Figure S6. Locations of transcription start sites (TSS) relative to HMRs in keratinocytes and fibroblasts. **(a, b)** Locations of TSSs (red points) relative to HMRs sorted by length in **(a)** fibroblasts HMRs within CGIs, **(b)** Fb-specific HMRs outside of CGIs. **(c, d)** same as in **(a, b)**, but showing TSS location with respect to keratinocytes HMRs. **(e)** Locations of TSSs relative to the common HMRs outside of CGIs.

Figure S7. Nucleosome organization at extended HMRs in keratinocytes. **(a)** Heatmap of CG methylation in keratinocytes (Ker) and fibroblasts (Fb) for the common HMRs in which the one boundary is identical, but the other boundary is extended in keratinocytes. The boundaries of these HMRs are grouped into three types: identical in two cells (boundary 1), not identical with short end (boundary 2), and with long end (boundary 3). These HMRs are aligned by boundary 2. **(b)** Heatmap of nucleosome positioning in keratinocytes and fibroblasts for the overlapping HMRs extended in fibroblasts aligned by boundary 2. **(c)** Average nucleosome density in fibroblasts (blue), keratinocytes (red), and intrinsic nucleosome occupancy scores (INOS) (black) for each boundary type of the extended HMRs.

Figure S8. Average nucleosome density and INOS at common HMRs with identical boundaries (C1). Average nucleosome occupancy measured in fibroblasts (blue) and keratinocytes (red) and intrinsic nucleosome occupancy scores (INOS) (black) in a 1,800 bp region (+/-900 bp) surrounding the **(a)** 5′ boundary and **(b)** the 3′ boundary of HMRs in which both boundary locations are identical in both cell types.

Figure S9. ROC curve of two predictors of nucleosome occupancy in fibroblast. **(a, b)** Receiver operating characteristic (ROC) curves of boundary CG (red) and intrinsic nucleosome occupancy scores (INOS) (black) for nucleosome occupancy in all HMRs **(a)** and fibroblast-specific HMRs of top 20% by relative nucleosome occupancy peak **(b)**. For each indicator, sensitivity (true positive rate) is plotted against 1-specificity (false positive rate). Accuracy is measured by the area under the ROC curve (AUC).

Figure S10. E-box motif enrichment at the boundaries of non-CGI HMRs in keratinocytes. **(a, b)** Comparison of 6-mer occurrences surrounding the **(a)** 5′ unmethylated CG **(b)** 3′ unmethylated

CG at the boundary of keratinocyte-specific HMRs in the top 20% and bottom 20% of *in vivo* nucleosome peaks. **(c)** Ratio of observed/expected frequencies of the CACGTG pattern at -5 to +5 CGs for keratinocyte HMRs (5′ CG): all HMRs (All, black circle), keratinocyte-specific HMRs (black square), and keratinocyte-specific HMRs with the top 20% *in vivo* nucleosome density at the boundary CG (Top 20%, black triangle). **(d)** Same as in **(c)** but at the 3′ boundary of keratinocyte HMRs (3′ CG).

Figure S1

Insert length distribution of MNase-seq libraries

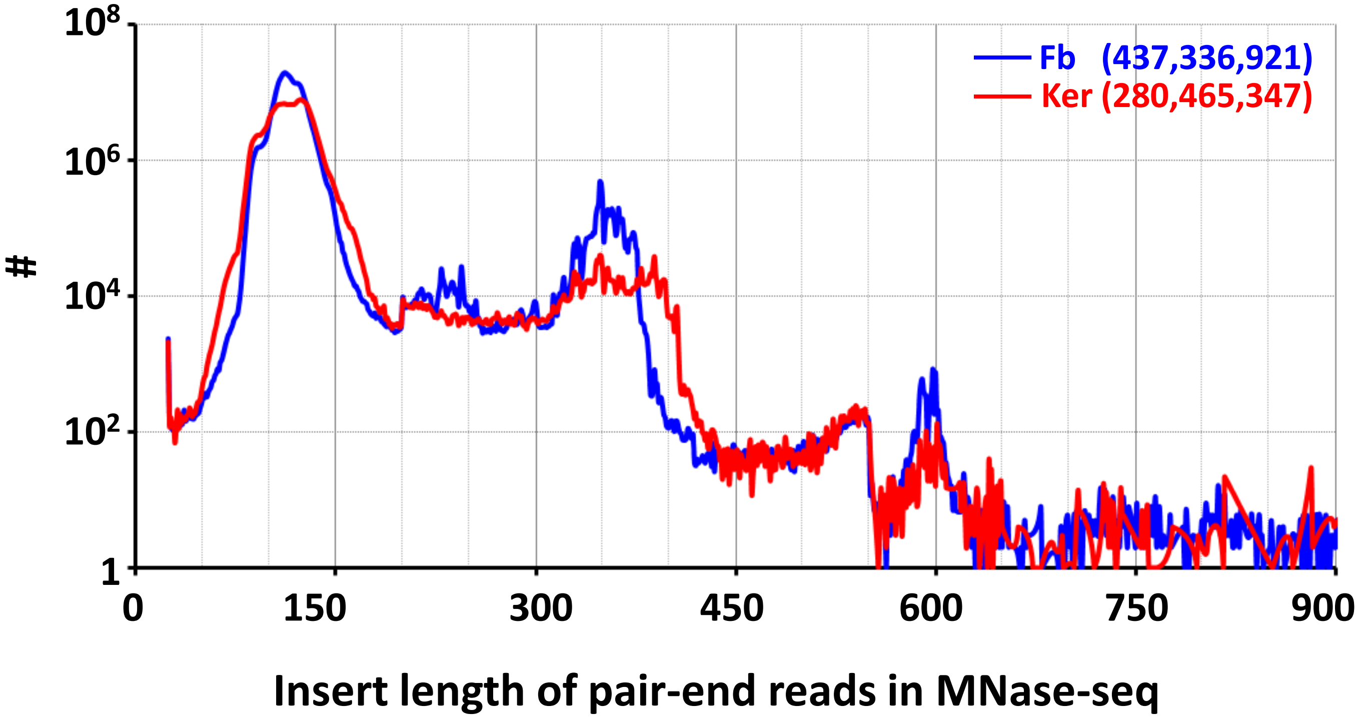

Figure S2

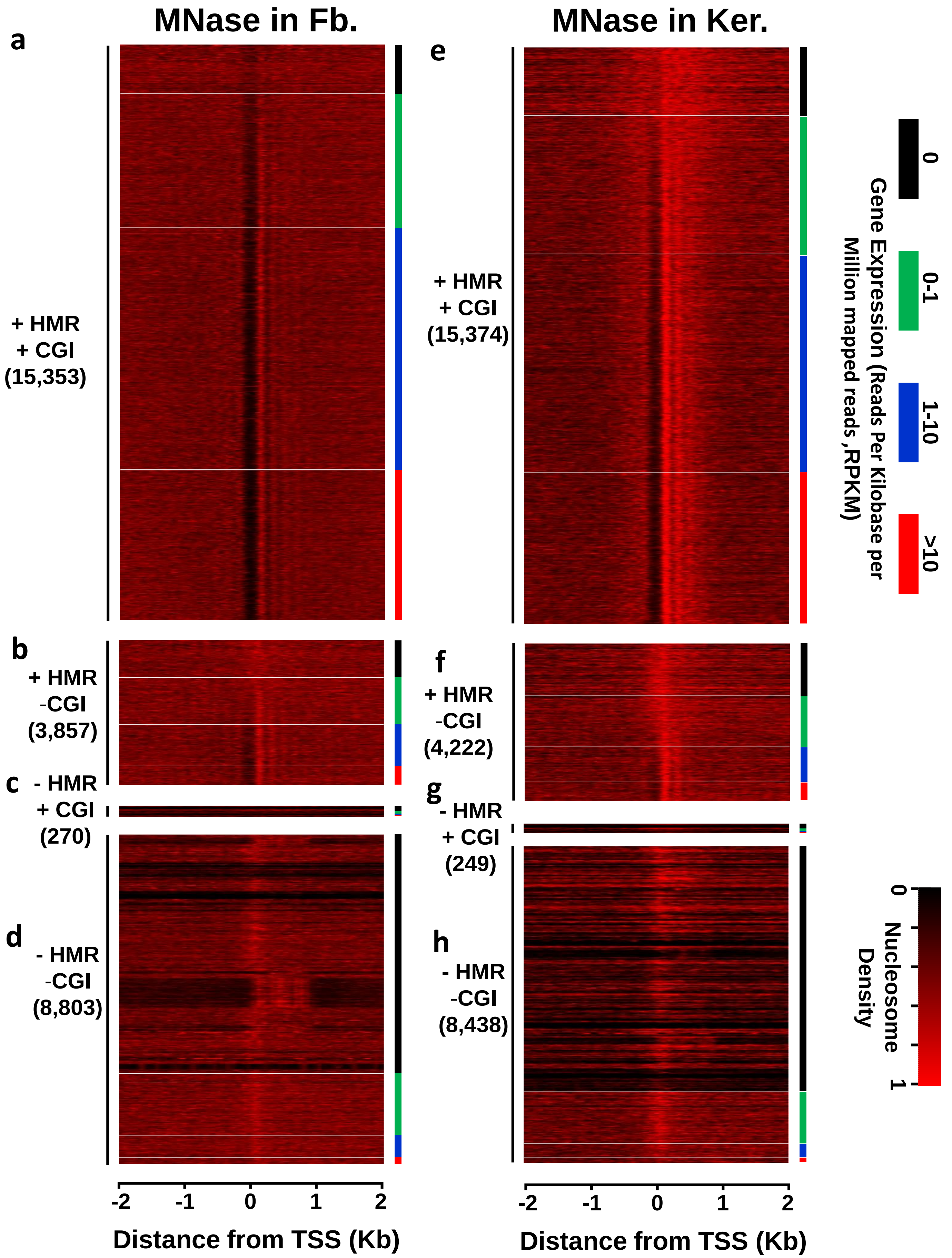
MNase in Fb.
MNase in Ker.
a
+ HMR
+ CGI
(15,353)
b
+ HMR
-CGI
(3,857)
c
- HMR
+ CGI
(270)
d
- HMR
-CGI
(8,803)
e
+ HMR
+ CGI
(15,374)
f
+ HMR
-CGI
(4,222)
g
- HMR
+ CGI
(249)
h
- HMR
-CGI
(8,438)
-2
-1
0
1
2
Distance from TSS (Kb)
Distance from TSS (Kb)
0
0-1
1-10
>10
Gene Expression (Reads Per Kilobase per Million mapped reads ,RPKM)
0
1
Nucleosome Density

Figure S3

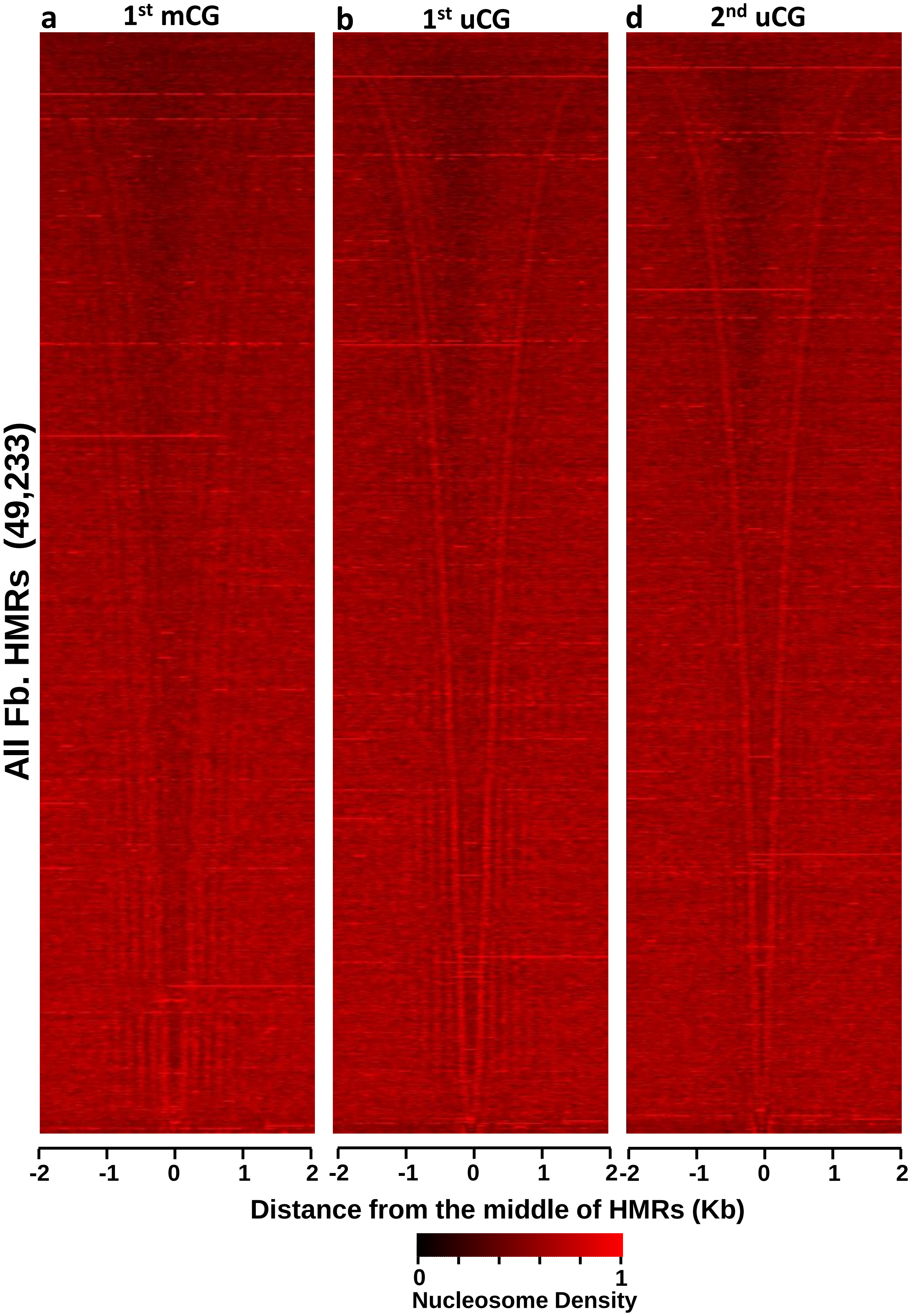
a
1st mCG
b
1st uCG
d
2nd uCG
All Fb. HMRs (49,233)
-2
-1
0
1
2
Distance from the middle of HMRs (Kb)
0
1
Nucleosome Density

Figure S4

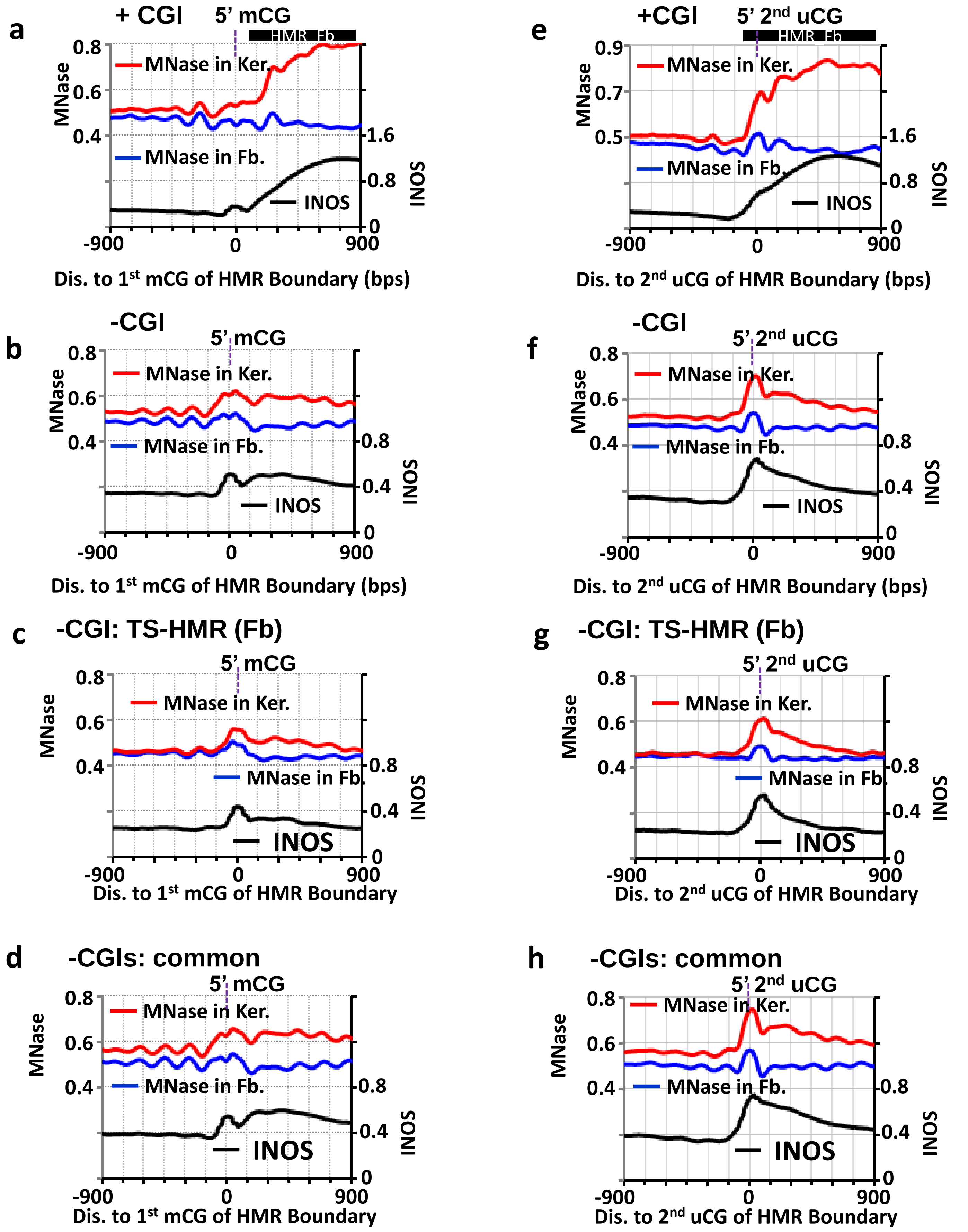

a
+ CGI
5' mCG
HMR_Fb
MNase in Ker.
MNase in Fb.
INOS
MNase
0.8
0.6
0.4
1.6
0.8
0
-900
0
900
Dis. to 1st mCG of HMR Boundary (bps)
b
-CGI
5' mCG
MNase in Ker.
MNase in Fb.
INOS
Dis. to 1st mCG of HMR Boundary (bps)
c
-CGI: TS-HMR (Fb)
5' mCG
MNase in Ker.
MNase in Fb.
INOS
Dis. to 1st mCG of HMR Boundary
d
-CGIs: common
5' mCG
MNase in Ker.
MNase in Fb.
INOS
Dis. to 1st mCG of HMR Boundary
e
+CGI
5' 2nd uCG
HMR_Fb
MNase in Ker.
MNase in Fb.
INOS
0.9
0.7
0.5
Dis. to 2nd uCG of HMR Boundary (bps)
f
-CGI
5' 2nd uCG
MNase in Ker.
MNase in Fb.
INOS
Dis. to 2nd uCG of HMR Boundary (bps)
g
-CGI: TS-HMR (Fb)
5' 2nd uCG
MNase in Ker.
MNase in Fb.
INOS
Dis. to 2nd uCG of HMR Boundary
h
-CGIs: common
5' 2nd uCG
MNase in Ker.
MNase in Fb.
INOS
Dis. to 2nd uCG of HMR Boundary

Figure S5

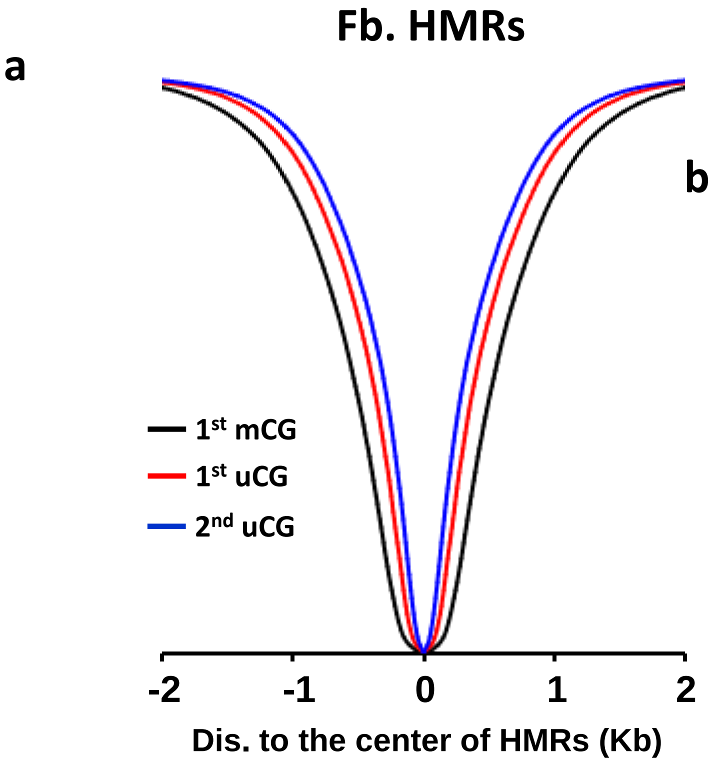
a
Fb. HMRs
1st mCG
1st uCG
2nd uCG
-2
-1
0
1
2
Dis. to the center of HMRs (Kb)

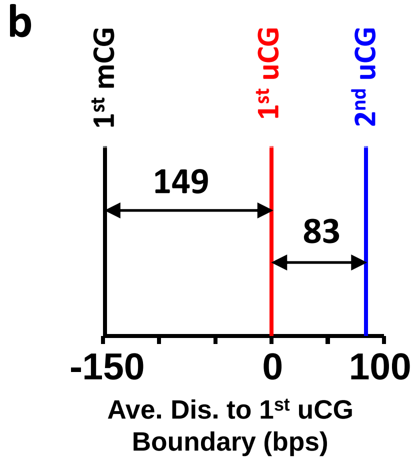
b
1st mCG
1st uCG
2nd uCG
149
83
-150
0
100
Ave. Dis. to 1st uCG Boundary (bps)

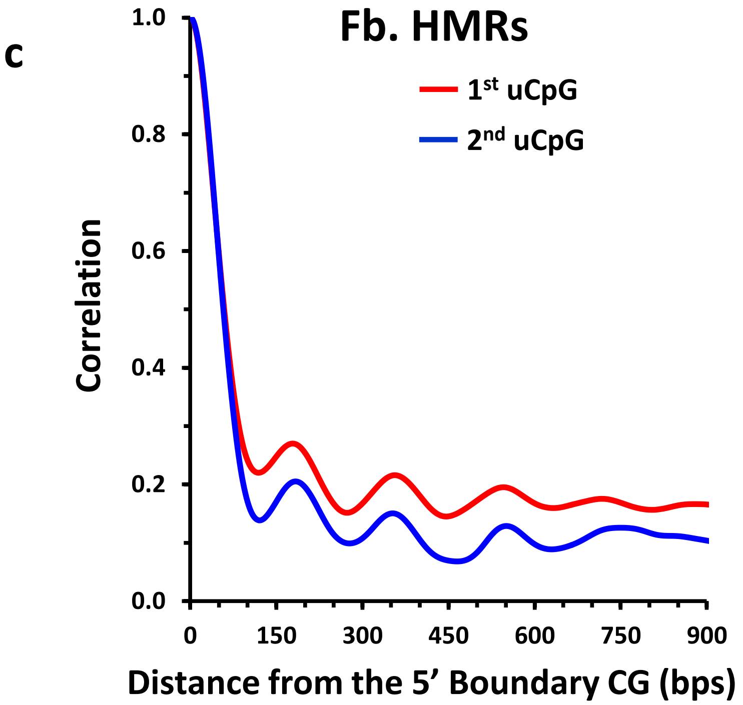
c
Fb. HMRs
1st uCpG
2nd uCpG
1.0
0.8
0.6
0.4
0.2
0.0
Correlation
0
150
300
450
600
750
900
Distance from the 5' Boundary CG (bps)

Figure S6

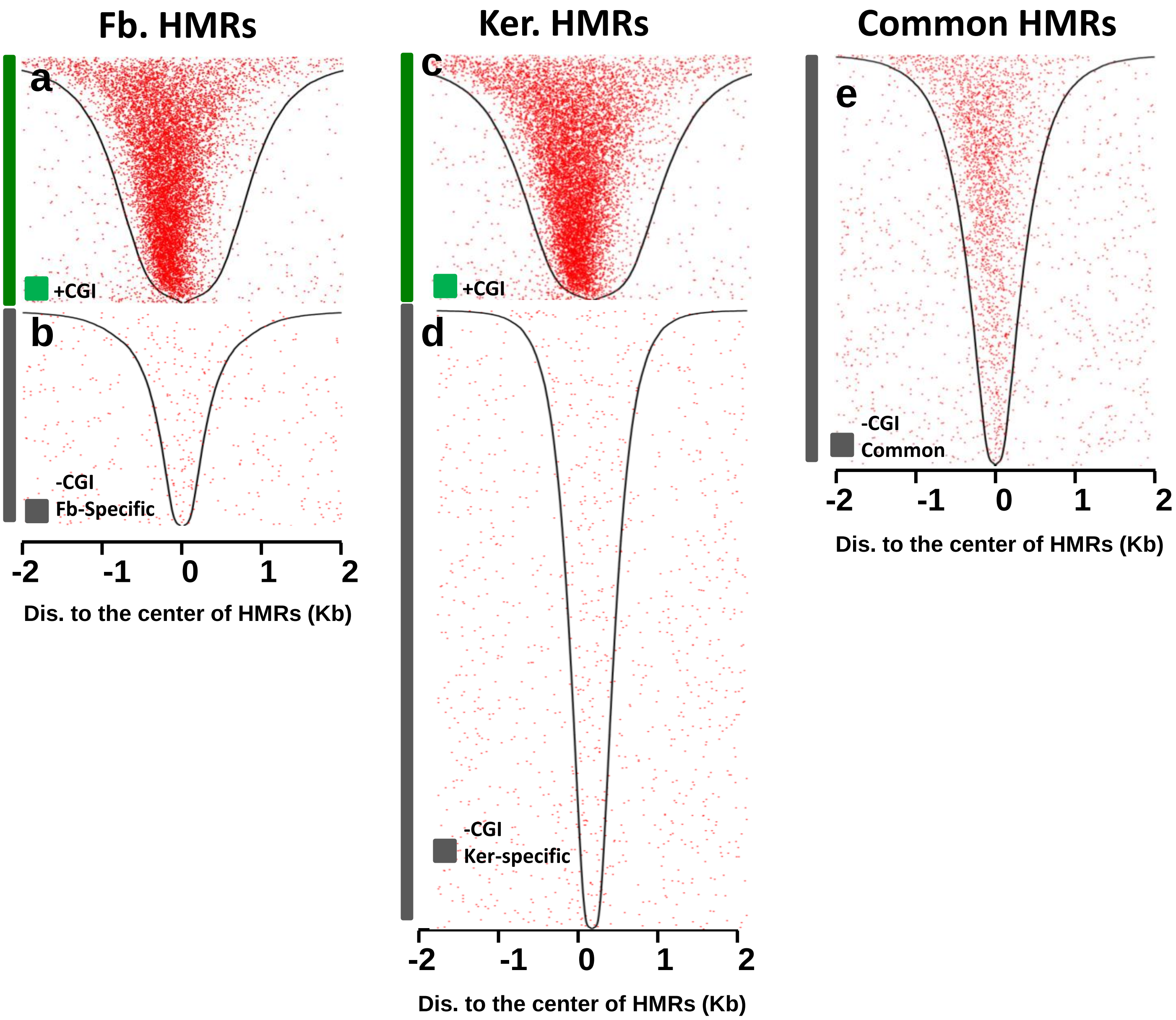

Fb. HMRs
Ker. HMRs
Common HMRs
a
+CGI
b
-CGI
Fb-Specific
c
+CGI
d
-CGI
Ker-specific
e
-CGI
Common
-2
-1
0
1
2
Dis. to the center of HMRs (Kb)

Figure S7

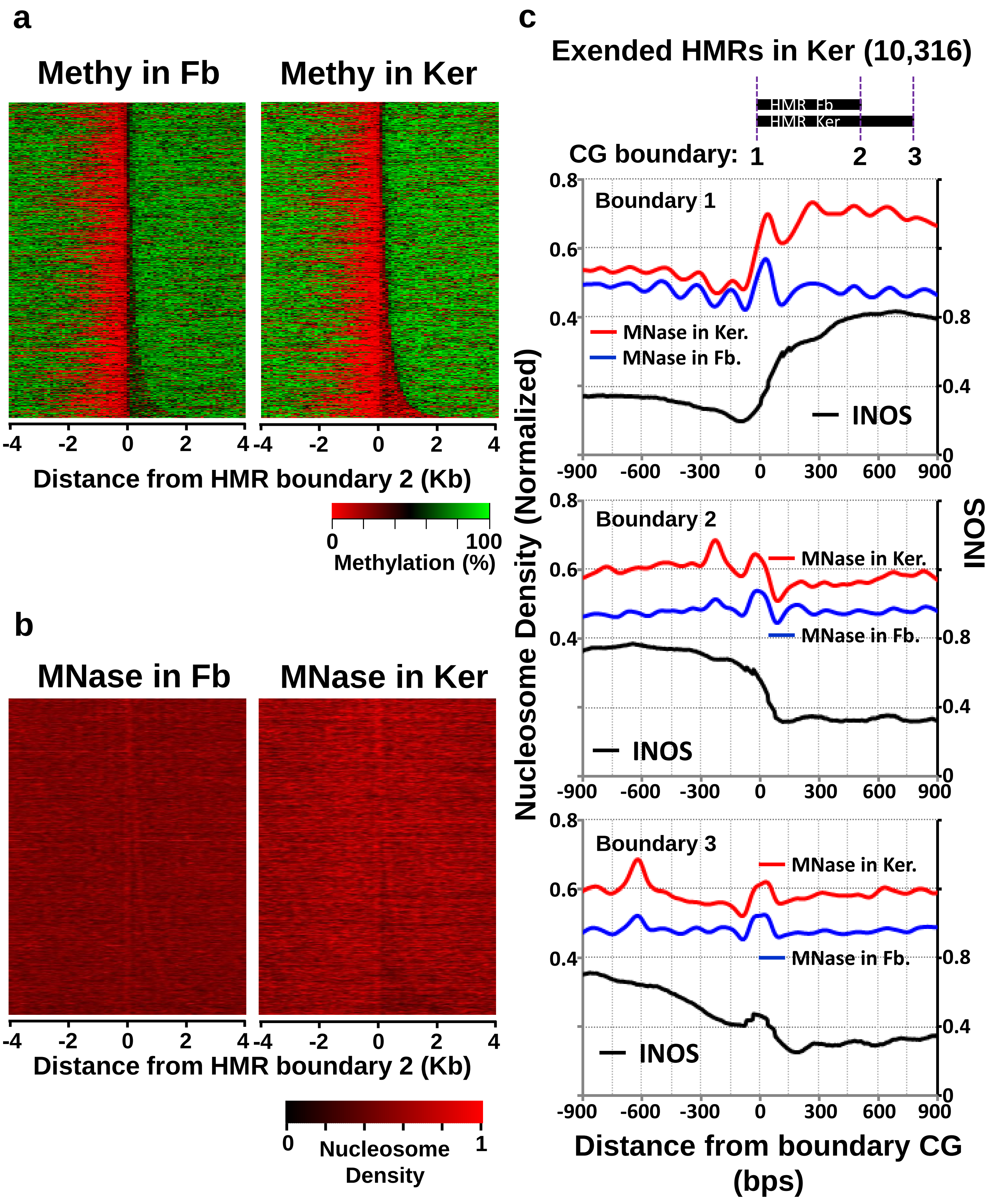

a
Methy in Fb
Methy in Ker
-4 -2 0 2 4
Distance from HMR boundary 2 (Kb)
0 100
Methylation (%)
b
MNase in Fb
MNase in Ker
Distance from HMR boundary 2 (Kb)
0 Nucleosome Density 1
c
Exended HMRs in Ker (10,316)
HMR_Fb
HMR_Ker
CG boundary: 1 2 3
Boundary 1
Boundary 2
Boundary 3
MNase in Ker.
MNase in Fb.
INOS
Nucleosome Density (Normalized)
INOS
0.8 0.6 0.4
0.8 0.4 0
-900 -600 -300 0 300 600 900
Distance from boundary CG (bps)

Figure S8

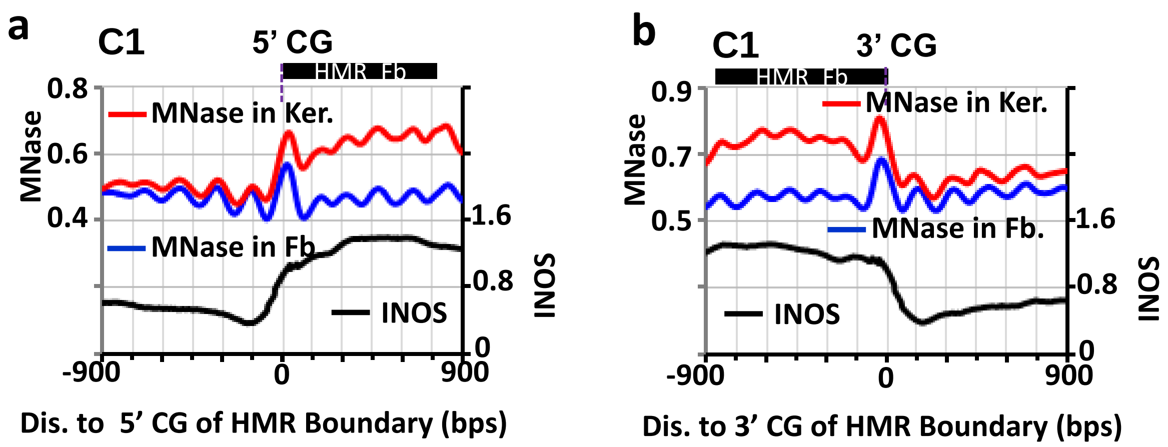
a
C1
5’ CG
HMR_Fb
MNase
0.8
0.6
0.4
MNase in Ker.
MNase in Fb
INOS
1.6
0.8
0
-900
0
900
Dis. to 5’ CG of HMR Boundary (bps)
b
C1
3’ CG
HMR_Fb
MNase
0.9
0.7
0.5
MNase in Ker.
MNase in Fb.
INOS
1.6
0.8
0
-900
0
900
Dis. to 3’ CG of HMR Boundary (bps)

Figure S9

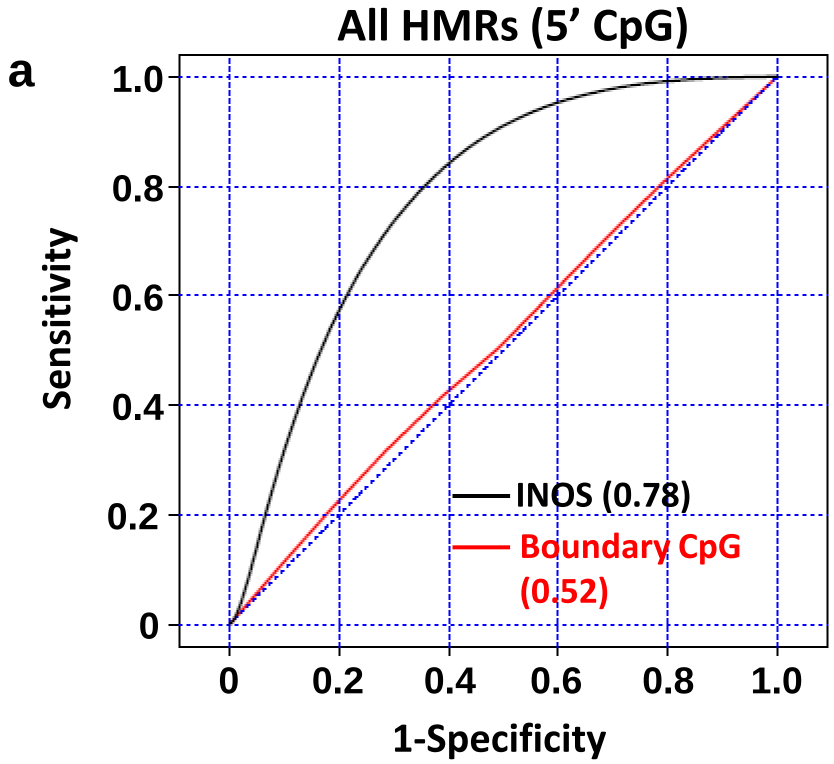
a
All HMRs (5’ CpG)
1.0
0.8
0.6
0.4
0.2
0
Sensitivity
0
0.2
0.4
0.6
0.8
1.0
1-Specificity
INOS (0.78)
Boundary CpG
(0.52)

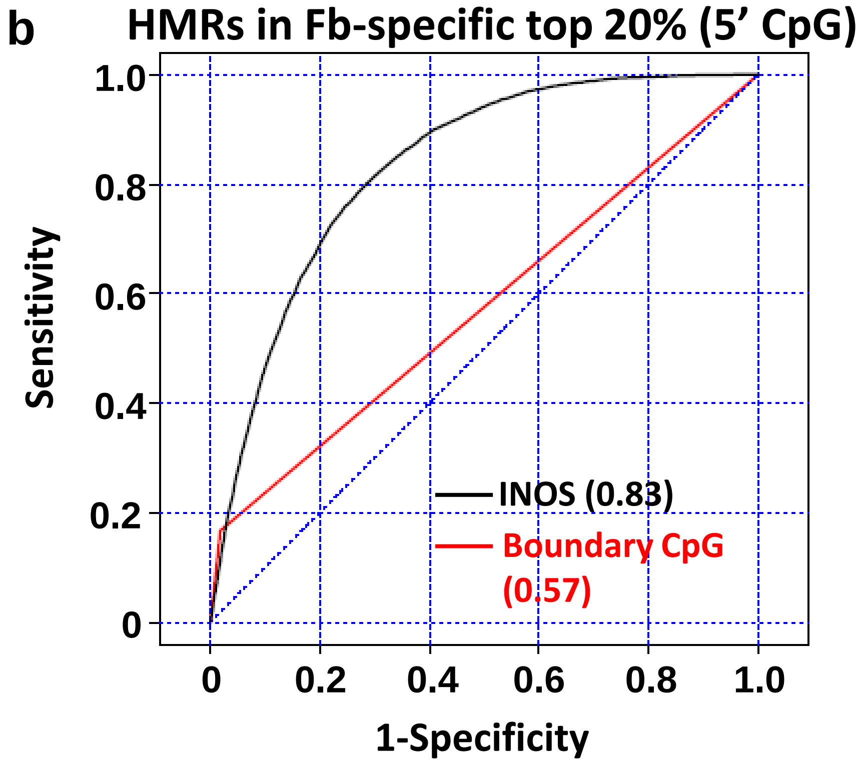
b
HMRs in Fb-specific top 20% (5’ CpG)
1.0
0.8
0.6
0.4
0.2
0
Sensitivity
0
0.2
0.4
0.6
0.8
1.0
1-Specificity
INOS (0.83)
Boundary CpG
(0.57)

Figure S10

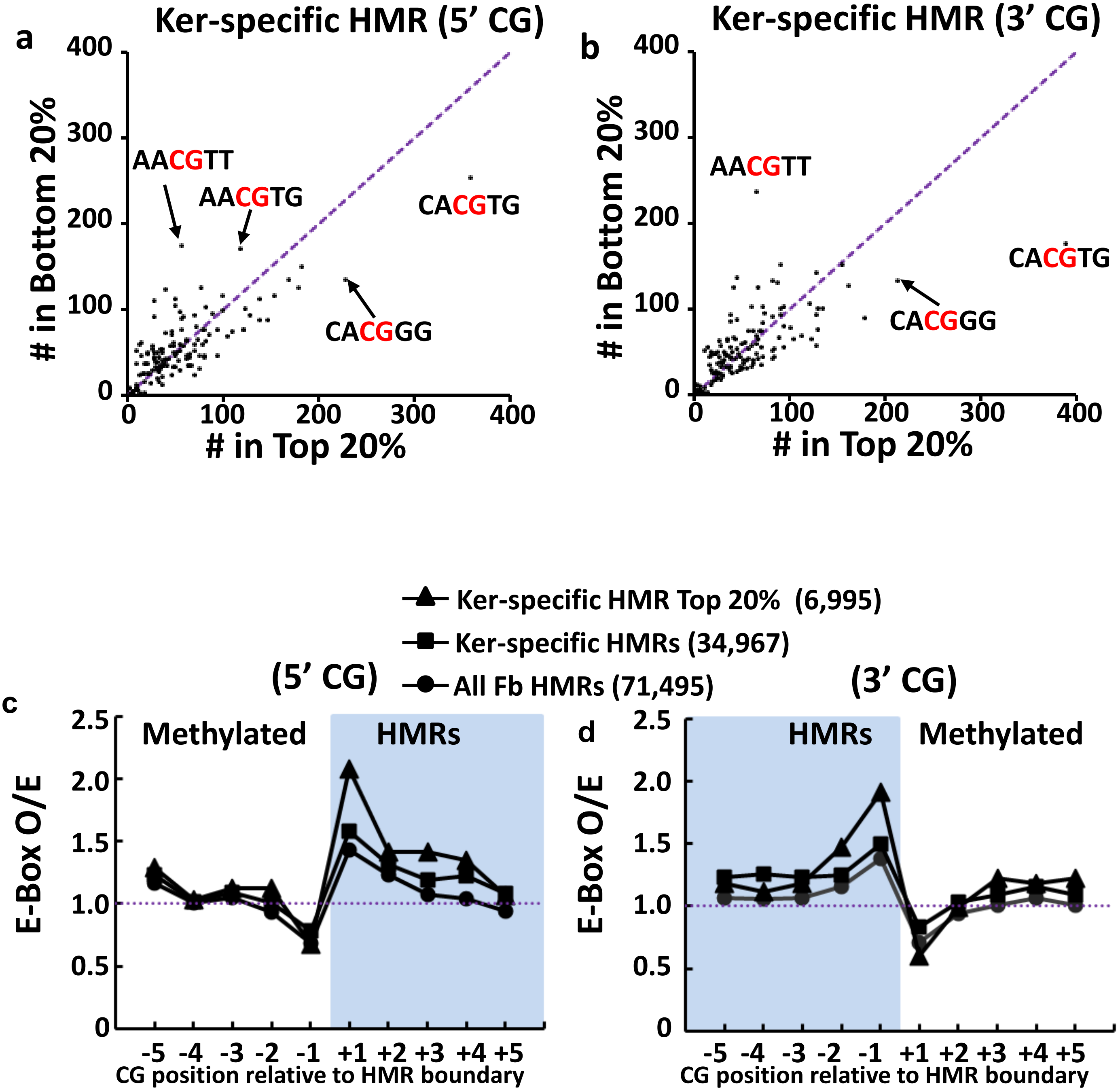
a
Ker-specific HMR (5' CG)
in Bottom 20%
in Top 20%
AACGTT
AACGTG
CACGTG
CACGGG
b
Ker-specific HMR (3' CG)
AACGTT
CACGTG
CACGGG
Ker-specific HMR Top 20% (6,995)
Ker-specific HMRs (34,967)
All Fb HMRs (71,495)
(5' CG)
(3' CG)
c
d
Methylated
HMRs
E-Box O/E
CG position relative to HMR boundary
